# Defining the Range of Pathogens Susceptible to Ifitm3 Restriction Using a Knockout Mouse Model

**DOI:** 10.1371/journal.pone.0080723

**Published:** 2013-11-21

**Authors:** Aaron R. Everitt, Simon Clare, Jacqueline U. McDonald, Leanne Kane, Katherine Harcourt, Malika Ahras, Amar Lall, Christine Hale, Angela Rodgers, Douglas B. Young, Ashraful Haque, Oliver Billker, John S. Tregoning, Gordon Dougan, Paul Kellam

**Affiliations:** 1 Wellcome Trust Sanger Institute, Wellcome Trust Genome Campus, Hinxton, United Kingdom; 2 Mucosal Infection and Immunity Group, Section of Infectious Diseases, Department of Medicine, Imperial College London, London, United Kingdom; 3 The Jenner Institute, University of Oxford, Oxford, United Kingdom; 4 Medical Research Council National Institute for Medical Research, London, United Kingdom; 5 Malaria Immunology Laboratory, Queensland Institute of Medical Research and The Australian Centre for Vaccine Development, Herston, Brisbane, Queensland, Australia; 6 Department of Infection, University College London, London, United Kingdom; Mayo Clinic, United States of America

## Abstract

The interferon-inducible transmembrane (IFITM) family of proteins has been shown to restrict a broad range of viruses *in vitro* and *in vivo* by halting progress through the late endosomal pathway. Further, single nucleotide polymorphisms (SNPs) in its sequence have been linked with risk of developing severe influenza virus infections in humans. The number of viruses restricted by this host protein has continued to grow since it was first demonstrated as playing an antiviral role; all of which enter cells via the endosomal pathway. We therefore sought to test the limits of antimicrobial restriction by Ifitm3 using a knockout mouse model. We showed that Ifitm3 does not impact on the restriction or pathogenesis of bacterial (*Salmonella* typhimurium, *Citrobacter rodentium*, *Mycobacterium tuberculosis*) or protozoan (*Plasmodium berghei*) pathogens, despite *in vitro* evidence. However, Ifitm3 is capable of restricting respiratory syncytial virus (RSV) *in vivo* either through directly restricting RSV cell infection, or by exerting a previously uncharacterised function controlling disease pathogenesis. This represents the first demonstration of a virus that enters directly through the plasma membrane, without the need for the endosomal pathway, being restricted by the IFITM family; therefore further defining the role of these antiviral proteins.

## Introduction

Intrinsic cellular defense molecules are able to detect and restrict invading pathogens at the level of the infected cell and constitute an initial repertoire of proteins that prevent infection. Such intrinsic defenses have the ability to detect the pathogen, and either directly block a component of the pathogen’s life cycles and / or signal to the innate and adaptive immune system to further control the infection. In certain cases, these intrinsic restriction factors recognise non-self pathogen-assocated molecular patterns, such as lipids, proteins and nucleic acids, from a broad range of pathogens through pathogen recognition receptors. This ability allows the host to detect bacterial, viral, fungal and protozoan pathogens [1]. For example, Toll-like receptor 4 alone is able to detect Gram-negative bacteria, fungi, trypanosomes and surface proteins on several viruses [2]. Alternatively, other restriction factors, particularly those that target viruses, appear to have a more reduced range of pathogens that they can block, as is the case for many interferon-stimulated genes (ISGs) [3]. However, restriction factors that work in defined cellular locations against a common pathogen feature of infection may also have broad anti-influenza properties.

We and others have found that the ISG interferon-inducible transmembrane 3 (IFITM3), initially defined as playing a developmental role in germ cell homing [4], has a profound role in the restriction of viruses entering the cell through the acid endosomal pathway [5,6], including influenza and dengue viruses [7]. Since the discovery of IFITM3’s antiviral role, the number of viruses restricted by the IFITM family has expanded considerably [5,7-14]. This has led to the generation of hypotheses about how the IFITM family achieves restriction; namely through preventing the fusion of viral and cellular membranes [15,16].

Recently, the role of IFITM3 has been expanded by the discovery of nonenveloped reoviruses’ restriction [9]. This has important implications, as nonenveloped viruses do not rely on membrane fusion to gain release from late endosomes. Instead, it is hypothesised that these viruses may physically disrupt the endosomal membrane through their surface proteins [17,18]. This therefore potentially broadens the actions of IFITM3 beyond enveloped viruses and may also include other non-viral pathogens.

The role of *IFITM3 in vivo* shows it is crucial restriction factor for preventing the onset of severe influenza viral infections in a knockout mouse model [6]. Further, the overrepresentation of a single nucleotide polymorphism (SNP), rs12252 C allele in the human *IFITM3* gene in two cohorts of patients hospitalised with influenza virus during the 2009 H1N1 pandemic shows that the rs12252 CC genotype confers an 4-5 fold increased risk developing a severe influenza virus infection [6,19].

Here we sought therefore to expand and define the role of Ifitm3 in pathogen restriction by assessing the susceptibility of Ifitm3-deficient (Ifitm3^-/-^) mice to bacteria (*Salmonella* Typhimurium, *Citrobacter rodentium*, *Mycobacterium tuberculosis*), a parasite (*Plasmodium berghei*) and a virus (respiratory syncytial virus, RSV) to determine the specificity of this crucial antimicrobial protein. We show that Ifitm3 is specifically an antiviral protein; yielding no significant phenotype in mice when challenged with bacteria and protozoa, despite studies implicating the IFITM family in restriction of these pathogens [20,21]. 

We also show a novel role for Ifitm3 *in vivo* in restriction of RSV: a virus that does not enter cells through the endosomal pathway, adding further to the role of IFITM3 as a central antiviral restriction factor that targets cellular entry.

## Methods

### Ethics statement

All animal experiments were conducted under Prof. Gordon Dougan’s project licence No. 80/2596, entitled “The characterization of microbial and murine genes required for infection”. This was brought before the Wellcome Trust Sanger Institute’s Animal Welfare and Ethical Review Body (formerly known as the Ethical Review Committee) on 30^th^ May 2012 and approved. The WTSI’s Animal Welfare and Ethical Review Body is constituted as required by the UK Animals (Scientific Procedures) Act 1986 Amendment Regulations 2012.

### Mice and general phenotyping

Background-matched 8-10 week old wild type, Ifitm3^-/-^ (Wellcome Trust Sanger Institute [22]) and Ifngr^-/-^ mice (Jackson Laboratories), all of which were >95% C57BL/6, were maintained in accordance with UK Home Office regulations, UK Animals Scientific Procedures Act 1986 under the project license PPL 80/2596. Animals were supplied with food and water *ad libitum* and were monitored daily for signs of illness. Ifitm3^-/-^ mice were phenotyped through pipelines at the Wellcome Trust Sanger Institute as described previously [23,24]. 

### Immunohistochemistry

5-µm sections of paraffin-embedded tissue were incubated with anti-Ifitm3 antibody (Abcam), which was subsequently bound to a secondary horse radish peroxidase-conjugated anti-rabbit antibody (Dako). Sections were counterstained with hematoxylin (Sigma-Aldrich) and were assessed for expression by microscopy.

### *Salmonella* Typhimurium challenge

Groups of 8 Ifitm3^-/-^ and 8 C57BL/6J mice were challenged intravenously with 5 x 10^5^ colony forming units (cfu) *Salmonella* Typhimurium M525 containing TetC, (Fragment C of tetanus toxin, to act as an antigen for subsequent antibody quantification), and followed for 28 days. On day 14 post-infection (pi), four from each group of mice are culled and organs (spleen, liver and caecum) removed. A small piece of the spleen and liver was fixed in 4% formalin and then later processed to paraffin blocks as a biobank of infected tissue for histological study if interesting. The rest of the organs were weighed then homogenized, serially diluted and plated to determine viable bacterial load. At day 28pi, the remaining four mice are culled following a terminal bleed and the organs removed and processed as above. The blood was allowed to clot then centrifuged when the serum was removed and used to detect TetC antigen specific antibodies by enzyme-linked immuosorbent assay (ELISA). Mice were weighed and monitored daily for signs of clinical illness.

### *Citrobacter rodentium* challenge

Groups of 8 Ifitm3^-/-^ and 8 C57BL6j mice were orally infected by gavage with 1 x 10^9^ cfu of and followed for 28 days. Every 2-3 days faeces from infected mice are collected. These were then weighed and homogenized in 1 ml per 100 mg of faeces. This was then serially diluted and plated to determine viable bacterial load. On day 14pi four mice per group were culled and organs (spleen, liver, caecal contents, caecum, 6cm of colon) removed. A small piece of the distal colon was fixed in 4% formalin and processed to paraffin blocks as a biobank of infected tissue for histological analysis. The rest of the organs were weighed then homogenized, serially diluted and plated to determine viable bacterial load. On day 28pi the remaining four mice were culled and the above is repeated. Mice were weighed and monitored daily for signs of clinical illness.

### *Mycobacterium tuberculosis* challenge

Mice were infected by low-dose aerosol exposure with H37Rv *M. tuberculosis* using a Glas-Col (Terre Haute, IN) aerosol generator calibrated to deliver approximately 100 bacteria into the lungs. Bacterial counts in the lungs at each time point of the study were determined by plating serial dilutions of individual lung homogenates on duplicate plates of Middlebrook 7H11 agar containing OADC enrichment. Colony-forming units were counted after 3–4 weeks incubation at 37 °C. 

### *Plasmodium* challenge

A transgenic *Plasmodium berghei* ANKA reporter line, PbGFP-LUC_CON_ (RMgm-28), that constitutively expresses a fusion protein of GFP and Firefly Luciferase [25], was maintained by passage in BALB/c mice. To induce experimental cerebral malaria (ECM), infected blood containing 5 x 10^5^ infected red blood cells was injected intraperitoneally. From day six pi mice were monitored twice daily and scored for clinical signs of neurologic disease using a ten parameter murine coma and behaviour scale adapted from that of Carroll et al. [26]. Mice classified as having ECM were killed by cervical dislocation. On days 2 and 3 pi parasite growth was monitored by luciferase measurements in 3 μl of blood, which were collected from a tail vein into Citrate-dextrose ACD freezing buffer (Sigma) and stored at -80°C until analysis using the Promega Bright-Glo Luciferase assay System with a Berthold Orion II microplate luminometer. Parasitemia was also monitored using Giemsa-stained thin blood smears. 

### Cytokine quantification

Plasma samples from *P. berghei* infected mice were analysed for cytokines using a Cytometric Bead Array Inflammation kit (BD Biosciences) according to manufacturer’s instructions.  Samples were acquired on a BD FACS Aria II, and data analysed using BD FCAP array software. 

### Respiratory syncytial virus challenge

RSV strain A2 (from Prof P. Openshaw, Imperial College London) was grown in HEp-2 cells and viral titer determined by plaque assay. Mice were infected intranasally (i.n.) with 5 x 10^5^ plaque forming units (PFU) under isoflurane anesthesia. Weight was measured daily to monitor disease severity. To collect bronchoalveolar lavage (BAL) fluid, the lungs of each mouse were inflated five times with 1 ml of PBS and BAL fluid kept on ice; 100 μl was centrifuged onto glass slides and stained with hematoxylin and eosin for cell differentiation. The remainder was centrifuged, the supernatant retained at −80°C, and the pellet resuspended in RPMI medium with 10% fetal calf serum. Lungs were removed, the smaller lobe was snap frozen in liquid nitrogen for RNA extraction and the remainder was homogenized by passage through 100-μm cell strainers (Falcon). Red blood cells in the lung sample were lysed in ammonium chloride buffer, and the remaining cells resuspended in RPMI medium with 10% fetal calf serum. Viable cell numbers were determined by trypan blue exclusion and lung cells types were differentiated by flow cytometry on a BD FACS Aria II using antibodies from BD and eBioscience. RSV viral load was measured by quantitative RT-PCR for the RSV L gene using primers and probes previously described [27], with L gene copy number determined using a RSV L gene standard and presented relative to μg lung RNA. Lungs were homogenised with a rotor-stator homogeniser, centrifuged and the supernatant collected for cytokine analyses. Cytokines in lung homogenate and BAL fluid were quantified using duosets from R&D systems. 

### Statistical analysis

All results are expressed as mean +/- S.D.; statistical significance was calculated by analysis of variance (ANOVA) followed by Bonferroni's Multiple Comparison Test when there were more than two groups and Student’s *t*-tests for the comparison of two groups. Non-normally distributed data were assessed by Mann-Whitney *U* test. Results regarding mouse survival were analysed by a Log-Rank (Mantel-Cox) test. All data regarding mouse phenotyping, including how individual traits were statistically analysed can be found at the Wellcome Trust Sanger Institute’s Mouse Resources Portal (http://www.sanger.ac.uk/mouseportal/) All calculations were performed using GraphPad Prism 5.0 software, and results were considered significant at p < 0.05.

## Results

### The role of Ifitm3 in murine homeostasis

**Figure 1:**
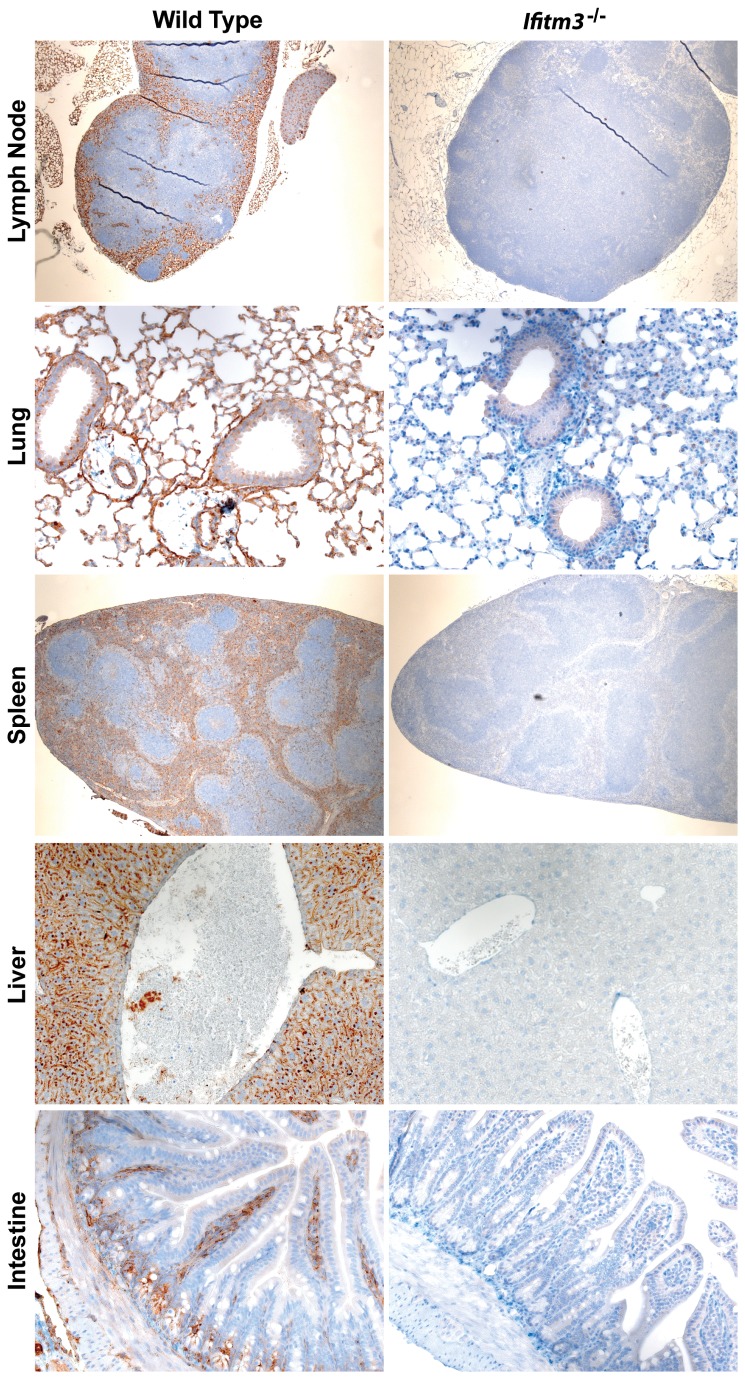
Expression of Ifitm3 at the predominant sites of pathogen infection. Paraffin-embedded sections from wild type and Ifitm3^-/-^ mice were cut and stained for expression of Ifitm3 (brown), and counterstained with hematoxylin (blue). Original magnification of lymph node and spleen 10×; lung and intestine 20×; liver 40×.

Many genes are embryonically lethal or lead to no overt phenotype when knocked out in mice. Indeed, Ifitm3^-/-^ mice have no discernable phenotype [22]. To examine in greater detail, uninfected mice were assessed against a panel of phenotypic assays [24], incorporating a robust set of adult traits that are capable of detecting phenotypic variations. We observed no statistically significant differences across all of our key phenotyping categories, including those assessing immune functions compared to wild type control mice (Table 1). Each of the categories listed in Table 1 can be further subdivided into a number of additional categories, again all being wild type in phenotype. The complete list of phenotyping can be accessed through the Wellcome Trust Sanger Institute’s Mouse Resources portal (http://www.sanger.ac.uk/mouseportal/). We assessed the tissue distribution of Ifitm3 by immunohistochemistry of wild type compared to Ifitm3^-/-^ mice, including lymph node, lung, spleen, liver and intestine. The expression of Ifitm3 was absent in all Ifitm3^-/-^ mouse organs, as expected, but was highly expressed in all wild type organs (Figure 1).

**Table 1:** Mammalian Phenotype (MP) ontology based heatmap for Ifitm3^-/-^ mice.

**MP category**		**MP category**		**MP category**		**MP category**	
Adipose tissue	NS	Endocrine / exocrine gland	NS	Immune system	NS	Pigmentation	NS
Behaviour / neurological	NS	Growth / size	NS	Integument	NS	Renal / urinary system	NS
Cardiovascular system	NS	Haematopoetic system	NS	Limbs / digits / tail	NS	Reproductive system	NS
Cellular	NS	Hearing / vestibular / ear	NS	Mortality / ageing	NS	Skeleton	NS
Craniofacial	NS	Homeostasis / metabolism	NS	Nervous system	NS		

The heatmap is built up of top-level terms from the MP ontology, with a second tier of tests included within each of the broader category headings listed in the table. Significant results that deviate from the “normal” murine phenotype are indicated by “S”, non-significant results are indicated by “NS”.

In wild type mice, the expression pattern of Ifitm3 was noteworthy. The spleen and lymph nodes indicated that Ifitm3 was predominantly expressed in the red pulp, but was absent from the white pulp. Similarly, intestinal staining revealed Ifitm3 expression to be high in the lamina propria, but not on the villus epithelium. Conversely, lung and liver showed ubiquitous expression of Ifitm3 throughout the tissues, with protein present in respiratory epithelial cells and hepatocytes, respectively.

As the first indication of the crucial role of IFITM3 only appeared upon infection with influenza [6] and the tissue distribution suggests Ifitm3 is important in multiple organ systems, we challenged the Ifitm3^-/-^ mice with a number of different pathogens.

### *Salmonella* Typhimurium challenge

**Figure 2:**
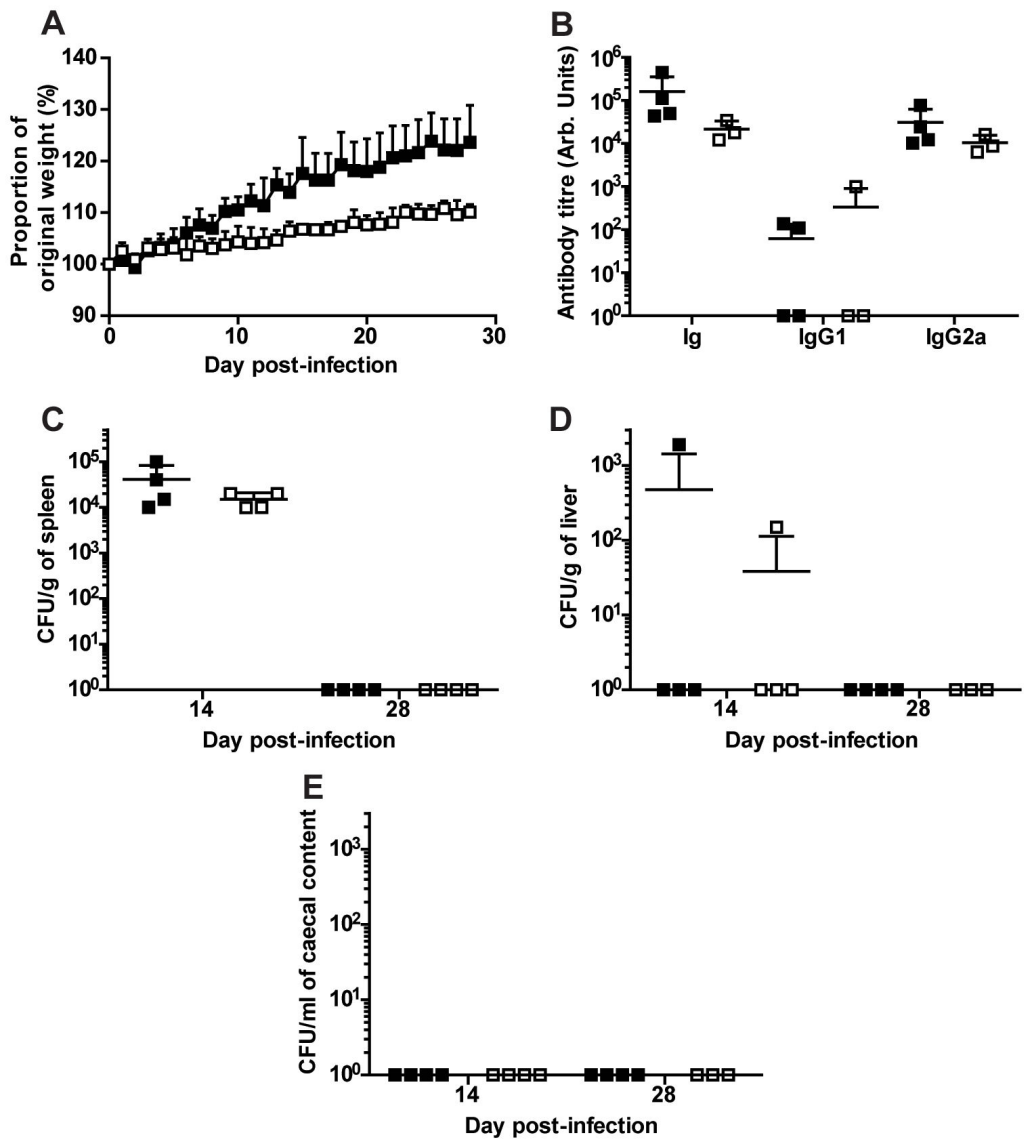
*Salmonella* Typhimurium challenge of wild type and Ifitm3^-/-^ mice. Mice were intravenously injected with *S*. Typhimurium and observed for weight loss for 28 days pi (**A**). Mice were killed on day 28 pi to assess neutralising antibody titre against *S*. Typhimurium (**B**). Bacterial contents from spleen (**C**), liver (**D**) and caecal contents (**E**) were titred on days 14 and 28 pi to assess the bacterial colonisation. ■: wild type, □: Ifitm3^-/-^. Results show means ± S.D. (n > 3).

Wild type and Ifitm3^-/-^ mice were intravenously dosed with 1 × 10^6^ CFU of *S*. Typhimurium M525 bacteria and observed for 28 days pi for signs of morbidity and weight loss (Figure 2A). All mice survived the challenge and gained weight over the time course of the study. Ifitm3^-/-^ mice appeared to gain proportional weight at a slower rate, however, this was due to these mice being on average 5g heavier at the start of challenge and based on lack of bacterial counts and pathology, is unlikely to be due to infection.

On day 28 pi, anti-*S*. Typhimurium antibody titres were determined from the sera of wild type and Ifitm3^-/-^ mice. This indicated that both genotypes of mice produced statistically similar antibody profiles (Figure 2B). Further, the bacterial load was determined in the spleen, liver and faecal contents (Figure 2C-E). Similarly, bacterial counts revealed no significant differences between wild type and Ifitm3^-/-^ mice; together showing that Ifitm3 does not play a role in resistance or susceptibility to *Salmonella* infection.

### *Citrobacter rodentium* challenge

**Figure 3:**
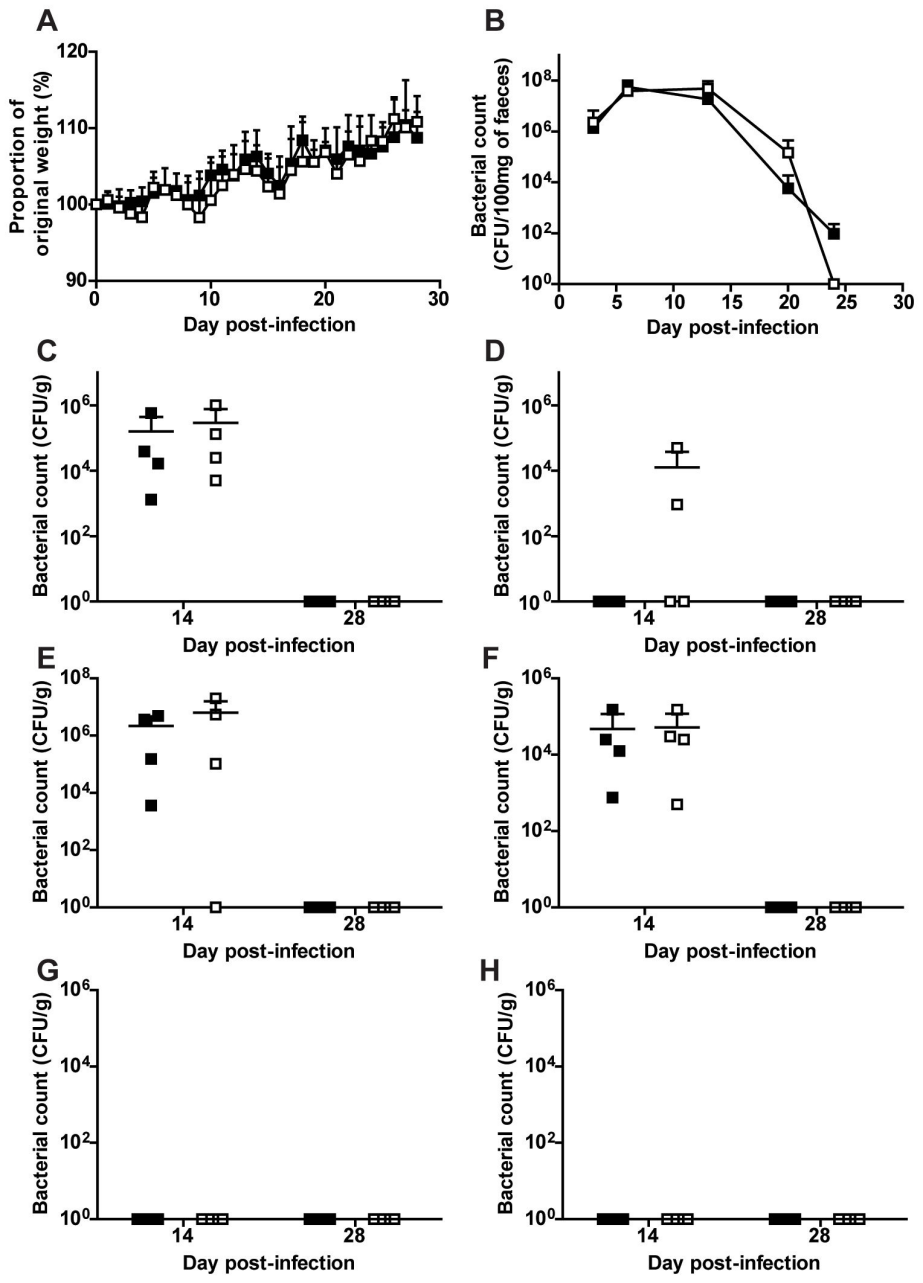
*Citrobacter rodentium* challenge of wild type and Ifitm3^-/-^ mice. Mice were orally infected with *C. rodentium* and weighed daily to monitor morbidity (**A**). Faecal samples were taken over the course of infection (**B**), and were homogenised, diluted and plated to count the number of colony forming units (CFU) shed over the course of the challenge. Mice were also killed on days 14 and 28 pi and CFU of *C. rodentium* were counted from caecal patch (**C**), caecum (**D**), colon (**E**), caecal contents (**F**), liver (**G**) and spleen (**H**). ■: wild type, □: Ifitm3^-/-^. Results show means ± S.D. (n > 4).

Wild type and Ifitm3^-/-^ mice were orally gavaged with 1 × 10^9^ CFU of C. rodentium bacteria and monitored for 28 days pi for signs of morbidity. Weight loss profiles showed that neither wild type nor Ifitm3^-/-^ mice had any overt signs of illness over the course of infection (Figure 3A). Bacteria shed in the faeces of these mice also revealed no significant differences between the genotypes, with clearance of infection achieved by day 25 pi in Ifitm3^-/-^ mice (Figure 3B). 

Mice were sacrificed on days 14 and 28 pi to determine any differences in the bacterial burden between wild type and Ifitm3^-/-^ mice. Counts in the caecum (total, caeceal patch and contents) and colon showed no significant differences in bacterial colonisation and clearance (Figure 3C-F). Similarly, analysis of the liver and spleen revealed no instances of bacteraemia in either wild type or Ifitm3^-/-^ mice (Figure 3G,H). Taken together, these data suggest Ifitm3 does not impact on *C. rodentium* infection or pathogenesis.

### *Mycobacterium tuberculosis* challenge

**Figure 4:**
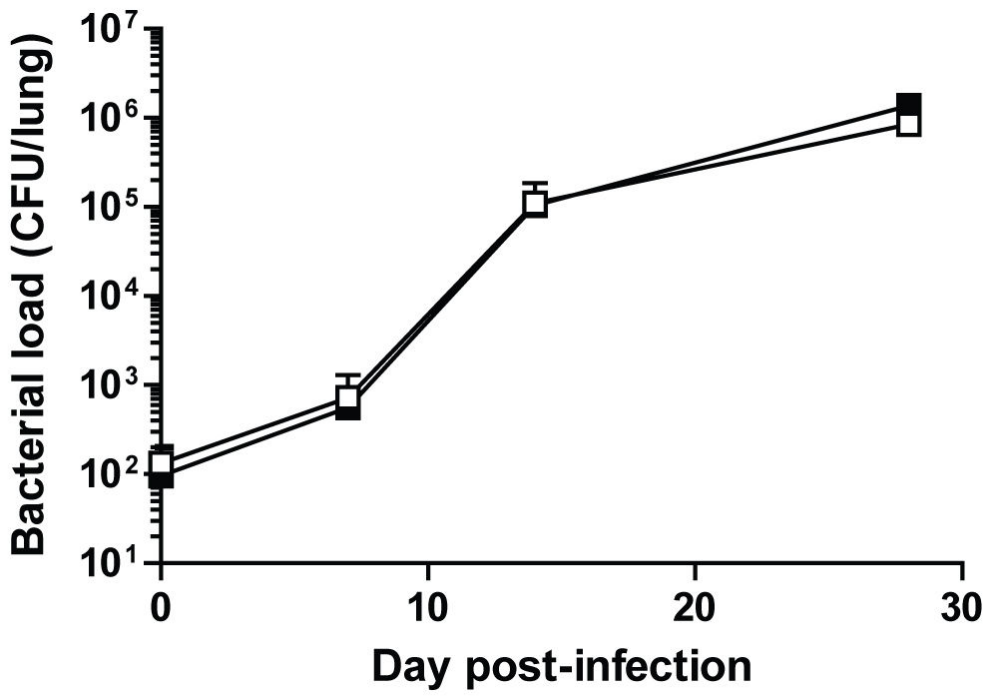
Bacterial growth kinetics of *M. tuberculosis* in the lungs of wild type and Ifitm3^-/-^ mice. Mice were killed over the course of infection with H37Rv *M. tuberculosis* to determine the bacterial load within their lungs. ■: wild type, □: Ifitm3^-/-^. Results show means ± S.D. (n > 5).

Wild type and Ifitm3^-/-^ mice were aerogenically infected with an aerosolised dose of approximately 100 CFU of H37Rv *M. tuberculosis* bacteria and monitored for signs of morbidity for the following 28 days. To determine whether Ifitm3 was involved in the control of the bacterial infection, mice were sacrificed on days 0, 7, 14 and 28 pi to calculate the bacterial burden in the lungs. There were however, no significant differences between wild type and Ifitm3^-/-^ mice (Figure 4), with bacterial growth kinetics indicating that Ifitm3 does not effect on *M. tuberculosis* infection and pathogenesis. 

### *Plasmodium berghei* challenge

**Figure 5:**
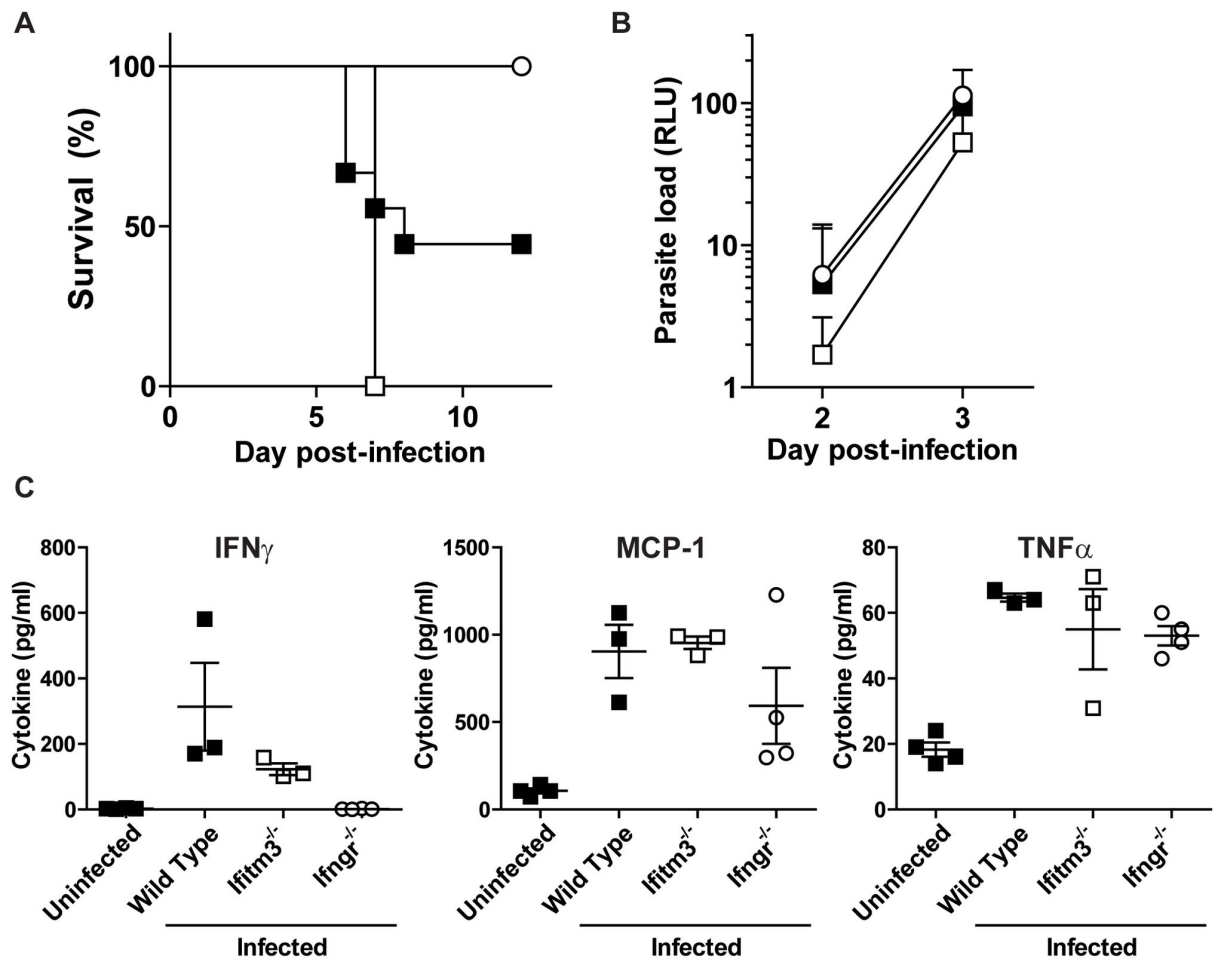
Malarial challenge of wild type and Ifitm3^-/-^ mice with *P. berghei* ANKA. Mice were intraperitoneally injected with red blood cells containing *P. berghei* ANKA and were monitored for survival for 12 days pi. n = 9 for wild type, n = 2 for each of Ifitm3^-/-^ and Ifngr^-/-^ mutants (**A**). Parasite biomass was monitored by measuring the activity of a luciferase reporter gene constitutively expressed by the parasite and expressed as relative light units (RLU) (**B**), and cytokine dysregulation was measured from the sera on day three pi by cytometric bead assay (**C**). ■: wild type, □: Ifitm3^-/-^, ○: Ifngr^-/-^. Results show means ± S.D. (n > 2).

Mice were intraperitoneally injected with 5 × 10^5^ red blood cells infected with a P. *berghei* ANKA reporter line, PbGFP-LUC_CON_ (RMgm-28), that constitutively expresses a fusion protein of GFP and Firefly Luciferase [25]. Interferon gamma (IFNγ) receptor knockout mice (Ifngr^-/-^) mice were included to act as control, as these mice do not succumb to lethal episodes of ECM. The experimental challenge revealed there to be no significant difference in phenotype seen in Ifitm3^-/-^ mice compared with wild type littermate controls, with both showing susceptibility to ECM (Figure 5A). The ~50% survival of wild type mice falls within acceptable boundaries owing to inherent inefficiencies in the delivery of parasites into the mice [28]. In contrast, Ifngr^-/-^ mice infected in parallel were fully protected from infection. Analysis of parasite burden revealed that all mice were infected with *P. berghei* (Figure 5B), but with no significant differences at day three pi. Additionally, levels of the inflammatory cytokines IFNγ, tumor necrosis factor alpha (TNFα) and monocyte chemotactic protein-1 (MCP-1) were analysed by cytometric bead array, again no significant differences between wild type and Ifitm3^-/-^ mice were observed (Figure 5C). 

### RSV challenge

Wild type and Ifitm3^-/-^ mice were intranasally infected with 5 × 10^5^ PFU of RSV-A (A2 strain) and were monitored daily for weight loss for seven days pi. Cohorts of mice were sacrificed on days four and seven pi to quantify viral burden and immunological changes over the course of the challenge.

**Figure 6:**
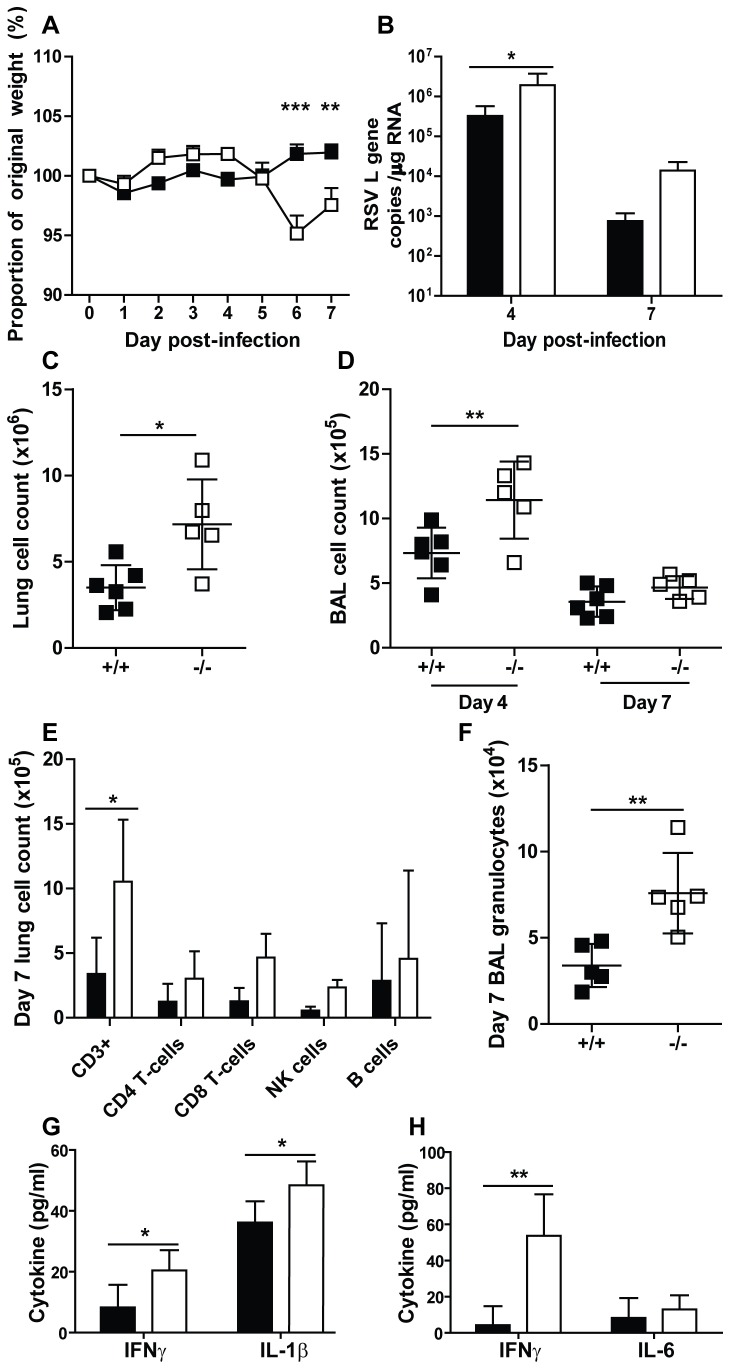
Comparison of RSV infection in Ifitm3^-/-^ mice to wild type mice. Ifitm3^-/-^ or wild type littermate control mice were infected i.n. with 5x10^5^ RSV A2. Mice were weighed daily and weight changes recorded as a percentage of original weight (**A**). Lungs were excised and viral load calculated by qPCR on days 4 and 7 pi (**B**). Total cell counts from lung (**C**) and BAL (**D**) were calculated, along with totals of CD3, CD4 and CD8 (T cells), CD19 (B cells) and DX5+ (NK cells) (**E**) measured in lung by flow cytometry on day 7 post infection. Granulocyte numbers were also calculated in the BAL on day 7 post infection (F) Levels of the inflammatory cytokines IFNγ and IL-1β in lung (**G**) and IFNγ and IL-6 in BAL (H) were measured by ELISA on day 7 post infection. ■: wild type, □: Ifitm3^-/-^. Results show means ± S.D. (n > 5). Statistical significance was assessed by Student’s *t*-test, or ANOVA followed by Bonferroni's Multiple Comparison Test when there were more than two groups (* *p*<0.05, ** *p*<0.01, *** p<0.001).

Ifitm3^-/-^ mice showed highly significant weight loss on days six and seven pi compared to wild type littermates (p < 0.01) (Figure 6A). Furthermore, Ifitm3^-/-^ mice showed a significantly higher peak in viral load on day four pi (p < 0.05), which remained higher than wild type mice at seven days pi (Figure 6B).

Bronchoalveolar lavage (BAL) was performed at days four and seven pi and lungs harvested at day seven for cell and cytokine measurements. Cellular infiltrate was quantified over the course of infection, which showed a significant increase in total cells resident in the lungs on day seven pi in Ifitm3^-/-^ mice (p < 0.05, Figure 6C) and a similarly significant increase in total cellular infiltrate in the BAL fluid on day four pi (p < 0.01, Figure 6D). Flow cytometry revealed an increase in all cellular sub-populations in Ifitm3^-/-^ mice relative to wild type littermates on day seven pi. In particular, numbers of total CD3+ T-cells (p < 0.05) in the lungs (Figure 6E) and granulocytes in the BAL fluid (p < 0.01, Figure 6F). Analysis of inflammatory cytokines, including IFNγ, IL-6 and IL-1β revealed differences in their levels between genotypes of mice in the lungs and BAL fluid on day seven pi (Figure 6G,H), with significantly higher levels of IFNγ (lung: p < 0.05, BAL: p < 0.05) and IL-1β (p < 0.05) in Ifitm3^-/-^ mice relative to wild type controls.

## Discussion

The role of IFITM3 in restricting virus infections, where the virus enters the cell through the acidic endosomal pathway, is well established [5,15,29]. However, IFITM3’s role in other infections or the effect in removing IFITM3 *in vivo* in the absence of infection is not well understood. Here we show Ifitm3 is expressed in many different murine tissues and cell types and does affect the response to RSV infection in mice. Ifitm3 does not contribute to the infection phenotype of *Citrobacter*, *Salmonella* or *Mycobacterium* bacterial infections. 

These bacterial species encompass a range of physiological and pathogenic niches. *Salmonella enterica* serovar Typhimurium (*S*. Typhimurium) is an intracellular bacteria that enters cells through phagocytosis or by a bacterial-triggered entry mechanism and replicates within endosomal-like structures known as *Salmonella*-containing vacuoles [30]. In contrast, *Citrobacter rodentium* (*C. rodentium*) is a non-invasive, Gram-negative bacterium used to model enteropathogenic and enterohaemorrhagic  *E. coli* infections of the gut [31,32], and *Mycobacterium tuberculosis* (*M. tuberculosis*) is an intracellular respiratory bacterium that replicates primarily within macrophages and dendritic cells, before forming latent granulomas in the infected organs [33], and is the causative agent of tuberculosis (TB). We found no evidence for control of *M. tuberculosis* bacterial growth in murine lungs, despite the fact that the pathogen triggers a type I IFN response [34], which subsequently up-regulates Ifitm3 expression. Further, a recent study implicated a SNP (rs3888188) in the promoter of *IFITM3* with susceptibility to TB [21], wherein the minority rs3888188-G allele was significantly overrepresented in patients with TB compared to healthy controls in a Han Chinese population. We found no evidence in our murine model that the ablation of Ifitm3 expression impacted on *M. tuberculosis* infection. However, it should be noted that we only assayed for initial infection and colonisation of the lungs and that the long term dormancy characteristic of human TB is not observed in the mouse model. Furthermore, these differences may have arisen due to our challenge strain differing from that used in the human study; therefore inducing different gene expression signatures.

Further, we found that Ifitm3 does not impact on the development of ECM in *Plasmodium berghei* ANKA infection, although it is well established that malarial parasites elicit strong type I and type II IFN responses in their hosts, which have been shown to impact on the severity of infection [35,36], with IFNα and IFNγ contributing to lethality in murine models. Furthermore, eight SNPs in the IFN receptor, *IFNAR1*, have been associated with the development of cerebral malaria in children; a finding that is corroborated in Ifnar^-/-^ mice, which also do not develop cerebral malaria [37]. *IFITM3*, along with several other ISGs, are significantly up-regulated in patients that have become infected with *P. falciparum* [20]. It was shown that deletion of several of these ISGs, including *Tbk1* and the double knockout of *Irf3* and *Irf7* prevented mice from developing lethal ECM [20,36]. Our work shows *IFITM3* is not an ISG involved in the pathogenesis of experimental cerebral malaria.

However, we saw that Ifitm3 was important in the control of RSV infection, leading to more severe disease in Ifitm3^-/-^ mice, as assessed by weight loss, viral load and a dysregulated immune response. Although these trends were seen with influenza virus infection of *Ifitm3* ^*-/-*^ mice, the phenotype seen in the RSV challenge is not as striking [6,38]. This may be due to the response to the different viruses and the genetic background of the mice (C57BL/6 Taconic), which we [39] and others [40] have shown is influential in RSV disease severity.

RSV is one of the commonest respiratory pathogens in children that necessitates hospitalisation [41]; accounting for three times more admissions to hospital than influenza viruses [42]. RSV, like influenza virus, is an enveloped virus that initially causes a mild upper respiratory tract infection that can develop into bronchiolitis and cause acute respiratory distress.

Our discovery that the lack of Ifitm3 can alter the pathogenesis of RSV infection suggests IFITM3 either directly restricts RSV cell infection *in vivo*, or exerts a hitherto uncharacterised function controlling virus infection *in vivo* is novel and supports associations seen in the mouse model [43,44], in airway epithelial cultures [45] and in blood from hospitalised infants [46]. Strikingly, RSV infects cells through the plasma membrane and does not require the endosomal pathway. RSV enters airway epithelial cells via F protein binding of nucleolin, which is situated in cholesterol rich microdomains / lipid rafts [47,48]. RSV is proposed to bind to nucleolin via its F protein, which initiates hemifusion of the RSV envelope with the cell membrane [48]; thus delivering the viral content directly into the cytoplasm without the need for endosomes.

Recently, Li and colleagues [49] suggested that the IFITM family of proteins are capable of restricting viral hemifusion and the formation of syncytia by reducing membrane fluidity [15,16]. Ifitm3, however, is primarily distributed intracellularly on endosomal membranes [50]. Of the Ifitm family members, Ifitm1 is primarily localised to the cell surface [15]: the site of RSV-cell fusion. Therefore Ifitm1, which is functional in the Ifitm3^-/-^ mice [6], may provide the strongest block to RSV infection. Previous studies have shown a degree of overlap of function between IFITM1, -2 and -3, with certain IFITMs having specificity for restricting particular viruses [7,8]. Importantly, IFITM1, -2 and -3 interact and may function co-operatively [15], possibly with IFITM3 potentiating an IFITM1 restriction of RSV. Indeed, *Ifitm1* has been shown to be up-regulated during RSV infection [51]. 

With the recent recognition of IFITM3-mediated restriction of nonenveloped reoviruses, the pleotropic effect of IFITM3 on diverse virus infections, or the co-operative role of all antiviral IFITM proteins as a layered defense to different virus infections, remains to be determined.
